# Supplementary material for: A SARS-CoV-2 antibody curbs viral nucleocapsid protein-induced complement hyperactivation
Source: Nat Commun. 2021 May 11;12:2697. doi: 10.1038/s41467-021-23036-9 (PMC8113585; doi:10.1038/s41467-021-23036-9)
Supplement: Supplementary file 1 — Supplementary Information [file 41467_2021_23036_MOESM1_ESM.pdf]

Supplementary information: A SARS-CoV-2 antibody curbs viral nucleocapsid protein-induced complement hyperactivation

Sisi Kang[1#], Mei Yang[1#], Suhua He[1#], Yueming Wang[2,3#], Xiaoxue Chen[1], Yao-Qing Chen[4], Zhongsi Hong[5], Jing Liu[6], Guanmin Jiang[7], Qiuyue Chen[1], Ziliang Zhou[1], Zhechong Zhou[1], Zhaoxia Huang[1], Xi Huang[8], Huanhuan He[1], Weihong Zheng[2,3], Hua-Xin Liao[2,3*], Fei Xiao[1,5*], Hong Shan[1,9*], Shoudeng Chen[1*]

1. Molecular Imaging Center, Guangdong Provincial Key Laboratory of Biomedical Imaging, The Fifth Affiliated Hospital, Sun Yat-sen University, Zhuhai, 519000, China
2. Institute of Biomedicine, Jinan University, Guangzhou, 510632, China
3. Zhuhai Trinomab Biotechnology Co., Ltd., Zhuhai, 519040, China
4. School of Public Health (Shenzhen), Sun Yat-sen University, Shenzhen, China
5. Department of Infectious Disease, The Fifth Affiliated Hospital, Sun Yat-sen University, Zhuhai, 519000, China
6. Department of Respiratory Disease, The Fifth Affiliated Hospital, Sun Yat-sen University, Zhuhai, 519000, China
7. Department of Clinical laboratory, The Fifth Affiliated Hospital of Sun Yat-sen University, Zhuhai, 519000, China
8. Center for Infection and Immunity, The Fifth Affiliated Hospital, Sun Yat-sen University, Zhuhai, 519000, China
9. Department of Intervention Medicine, The Fifth Affiliated Hospital, Sun Yat-sen University, Zhuhai, 519000, China

# These authors contributed equally.

* Correspondence: Shoudeng Chen (Lead Contact: chenshd5@mail.sysu.edu.cn); Hong Shan (shanhong@mail.sysu.edu.cn); Fei Xiao (xiaof35@mail.sysu.edu.cn); Hua-Xin Liao (tliao805@jnu.edu.cn)

Supplementary information

Supplementary Figures 1-9

Supplementary Tables 1-11, 13

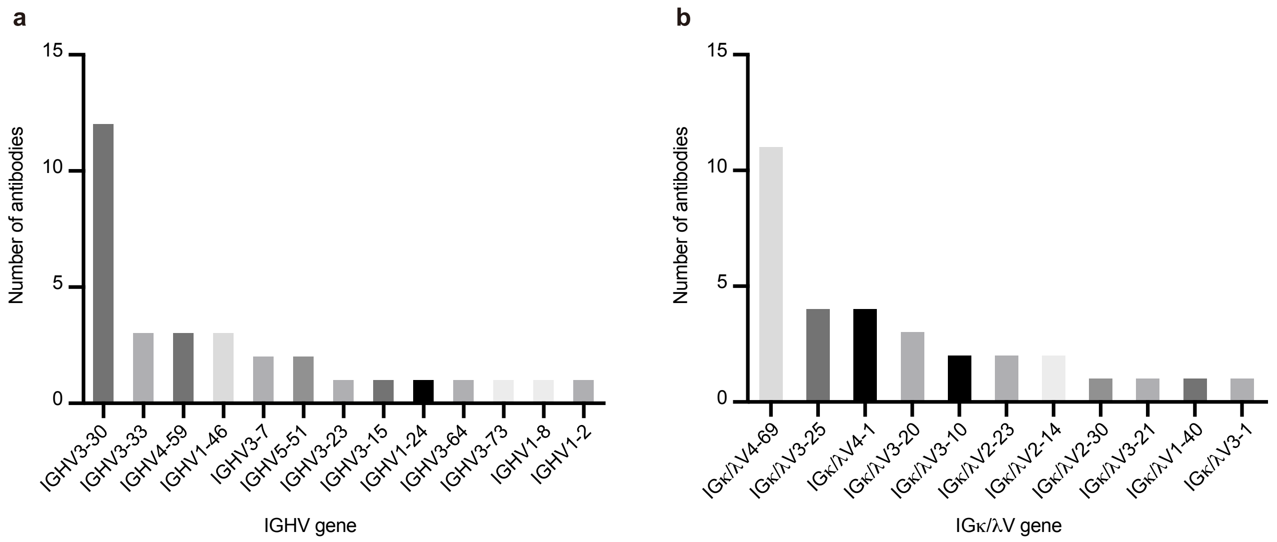

Supplementary Figure 1 | The distribution of IGHV gene and IGκ/λV gene usage of SARS-CoV-2 N-reactive antibodies. A total of 32 SARS-CoV-2 N-reactive antibodies from ZD006 were analyzed. (**a**) Number of IGHV gene from 32 antibodies in this study is shown on the Y-axis, IGHV gene is indicated on the X-axis. (**b**) Number of IGκ/λV gene from 32 antibodies in this study is shown on the Y-axis, IGκ/λV gene is indicated on the X-axis.

a nCoV396 Variable Domain

>L

```
  1 QLVLTQSPSASASLGASVKLTCTLSSGHSNYAIAWHQQQPEKGPRYLMKVNSDGSHTKGD   60
 61 GIPDRFSGSSSGAERYLTISSLQSEDEADYYCQTWGTGIQVFGGGTKLTVLGQPKAAPSV  120
121 TLFPPSSEELQANKATLVCLISDFYPGAVTVAWKADSSPVKAGVETTTPSKQSNNKYAAS  180
181 SYLSLTPEQWKSHRSYSCQVTHEGSTVEKTVAPTECS
```

>H

```
  1 QVQLVESGGGVVQPGRSLRLSCAASGFTFSSYIMHWVRQAPGKGLEWVAVISYDGSNEAY   60
 61 ADSVKGRFTISRDNSKNTLYLQMSSLRAEDTGVYYCARETGDYSSSWYDSWGRGTLVTVS  120
121 SASTKGPSVFPLAPSSKSTSGGTAALGCLVKDYFPEPVTVSWNSGALTSGVHTFPAVLQS  180
181 SGLYSLSSVVTVPSSSLGTQTYICNVNHKPSNTKVDKRVEPKSCDK
```

b nCoV396 Numbering

L Light Chain

Q	L	V	L	T	Q	S	P	S	A	S	A	S	L	G	A	S	V	K	L
1	2	3	4	5	6	7	8	9	11	12	13	14	15	16	17	18	19	20	21

T	C	T	L	S	S	G	H	S	N	Y	A	I	A	W	H	Q	Q	Q	P
22	23	24	25	26	27	28	29	30	30A	31	32	33	34	35	36	37	38	39	40

E	K	G	P	R	Y	L	M	K	V	N	S	D	G	S	H	T	K	G	D
41	42	43	44	45	46	47	48	49	50	51	52	53	54	54A	54B	54C	54D	55	56

G	I	P	D	R	F	S	G	S	S	S	G	A	E	R	Y	L	T	I	S
57	58	59	60	61	62	63	64	65	66	67	68	69	70	71	72	73	74	75	76

S	L	Q	S	E	D	E	A	D	Y	Y	C	Q	T	W	G	T	G	I	Q
77	78	79	80	81	82	83	84	85	86	87	88	89	90	91	92	93	94	95	96

V	F	G	G	G	T	K	L	T	V	L	G	Q	P	K	A
97	98	99	100	101	102	103	104	105	106	106A	107	108	109	110	111

H Heavy Chain

Q	V	Q	L	V	E	S	G	G	G	V	V	Q	P	G	R	S	L	R	L
1	2	3	4	5	6	7	8	9	10	11	12	13	14	15	16	17	18	19	20

S	C	A	A	S	G	F	T	F	S	S	Y	I	M	H	W	V	R	Q	A
21	22	23	24	25	26	27	28	29	30	31	32	33	34	35	36	37	38	39	40

P	G	K	G	L	E	W	V	A	V	I	S	Y	D	G	S	N	E	A	Y
41	42	43	44	45	46	47	48	49	50	51	52	52A	53	54	55	56	57	58	59

A	D	S	V	K	G	R	F	T	I	S	R	D	N	S	K	N	T	L	Y
60	61	62	63	64	65	66	67	68	69	70	71	72	73	74	75	76	77	78	79

L	Q	M	S	S	L	R	A	E	D	T	G	V	Y	Y	C	A	R	E	T
80	81	82	82A	82B	82C	83	84	85	86	87	88	89	90	91	92	93	94	95	96

G	D	Y	S	S	S	W	Y	D	S	W	G	R	G	T	L	V	T	V	S
97	98	99	100	100A	100B	100C	100D	101	102	103	104	105	106	107	108	109	110	111	112

S	A
113	114

Supplementary Figure 2 | CDRs delimitation and Kabat nomenclature of the mAb nCoV396. (**a**) CDRs are highlighted in colors (CDR1(red), CDR2(orange), CDR3(green)). The gray letters indicate the non-variable-domain region. The underlined black letters indicate variable domain of heavy chain while the other black letters indicate variable

domain of light chain. (**b**) The CDRs are highlighted in pink (CDR1, CDR2, CDR3). The green letters indicate heavy chain region while the blue letters indicate light chain region.

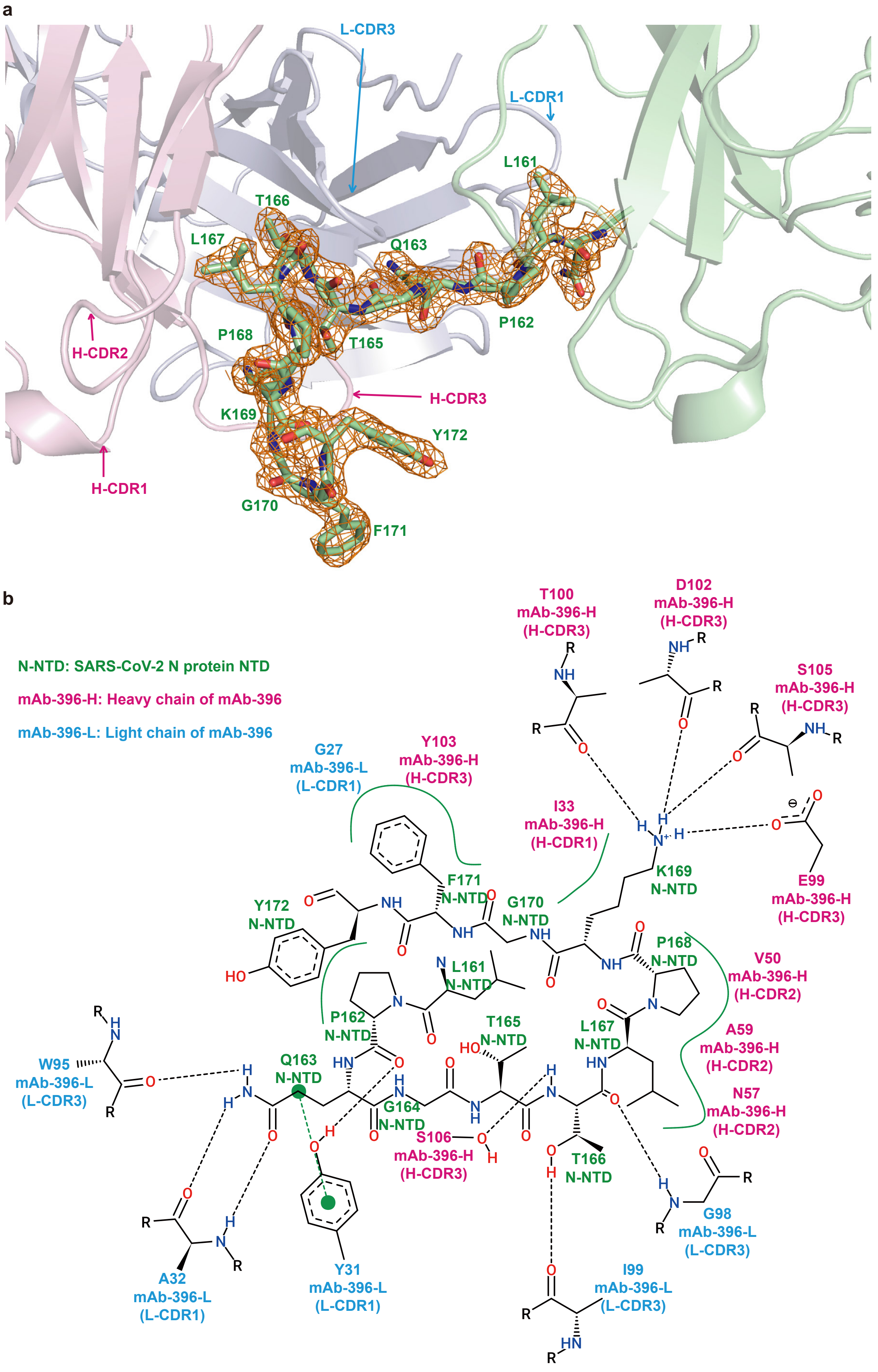
a
L-CDR3
L-CDR1
L161
T166
L167
Q163
P162
P168
T165
H-CDR2
H-CDR3
K169
Y172
H-CDR1
G170
F171
b
N-NTD: SARS-CoV-2 N protein NTD
mAb-396-H: Heavy chain of mAb-396
mAb-396-L: Light chain of mAb-396
T100 mAb-396-H (H-CDR3)
D102 mAb-396-H (H-CDR3)
S105 mAb-396-H (H-CDR3)
G27 mAb-396-L (L-CDR1)
Y103 mAb-396-H (H-CDR3)
I33 mAb-396-H (H-CDR1)
E99 mAb-396-H (H-CDR3)
K169 N-NTD
F171 N-NTD
G170 N-NTD
Y172 N-NTD
P168 N-NTD
L161 N-NTD
V50 mAb-396-H (H-CDR2)
P162 N-NTD
T165 N-NTD
L167 N-NTD
A59 mAb-396-H (H-CDR2)
W95 mAb-396-L (L-CDR3)
Q163 N-NTD
G164 N-NTD
N57 mAb-396-H (H-CDR2)
S106 mAb-396-H (H-CDR3)
T166 N-NTD
G98 mAb-396-L (L-CDR3)
A32 mAb-396-L (L-CDR1)
Y31 mAb-396-L (L-CDR1)
I99 mAb-396-L (L-CDR3)

Supplementary Figure 3 | Epitope interacting mechanism of mAb nCoV396 with SARS-CoV-2 N-NTD complex structure. (**a**) 2Fo-Fc maps (1.2σ level) for residues involved in intermolecular contacts. Epitope of SARS-CoV-2 N-NTD is illustrated with green colored stick representation, with 2Fo-Fc maps (1.2σ level). (**b**) Detail binding characterizations of involving residues in the complex. Residues in red color belong to heavy chain of mAb nCoV396, in blue color belong to light chain of mAb nCoV396, and in green color belong to N-NTD. Hydrogen bond is represented with black dash lines, while hydrophobic interaction is represented with green curves wrapped around the residues. The figure is generated by PoseView.

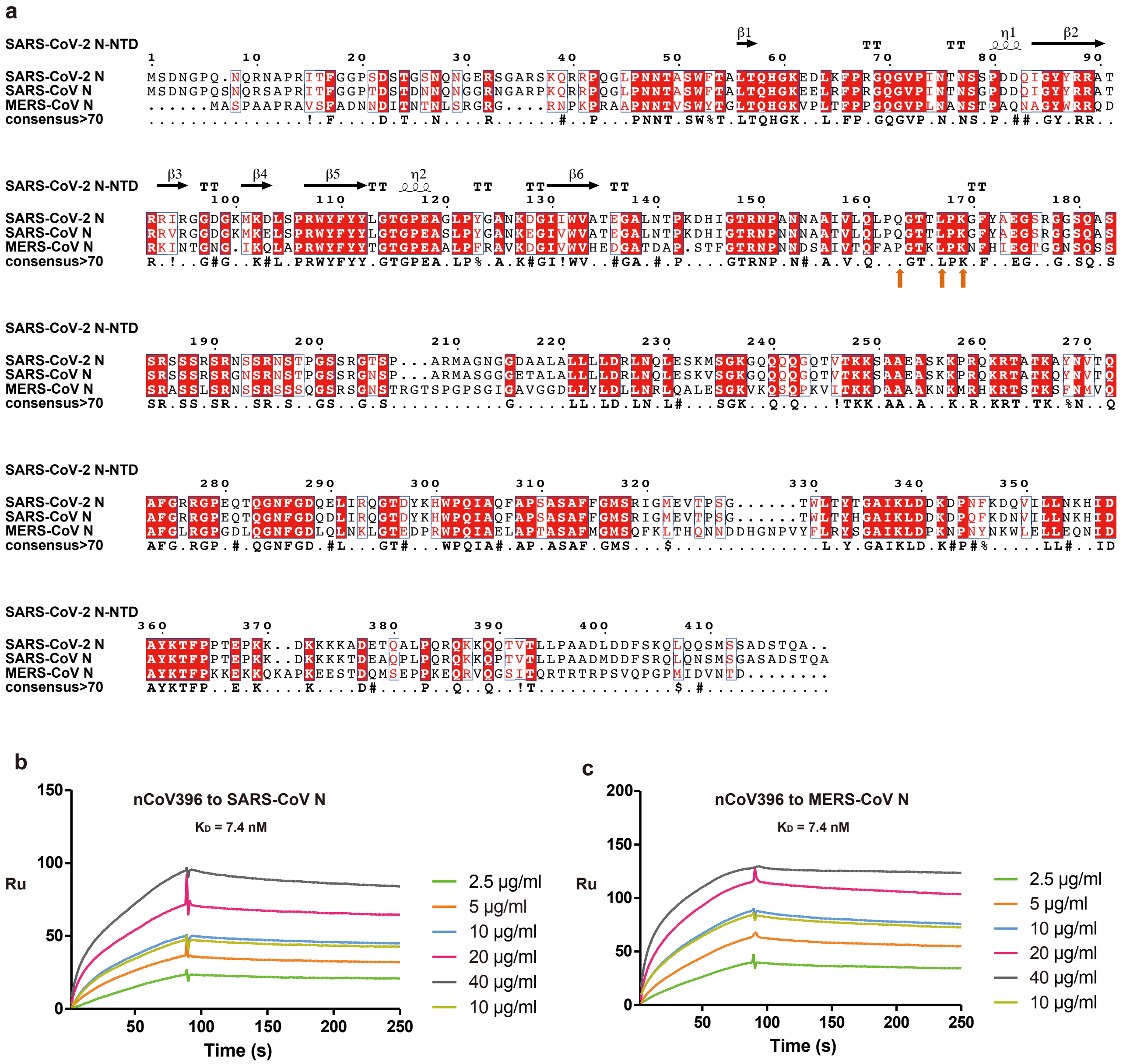

Supplementary Figure 4 | Binding of mAb nCoV396 to other coronavirus N proteins. (**a**) Sequence alignment of highly pathogenic N protein (SARS-CoV-2 N, SARS-CoV N and MERS-CoV N). The η symbol refers to a 3_{10}-helix. β-strands are rendered as arrows, strict β-turns as TT letters. The indicated arrows are the conserved residues recognized by mAb nCoV396. SPR binding affinity measurements of mAb nCoV396 to SARS-CoV N protein (**b**) and MERS-CoV N protein (**c**).

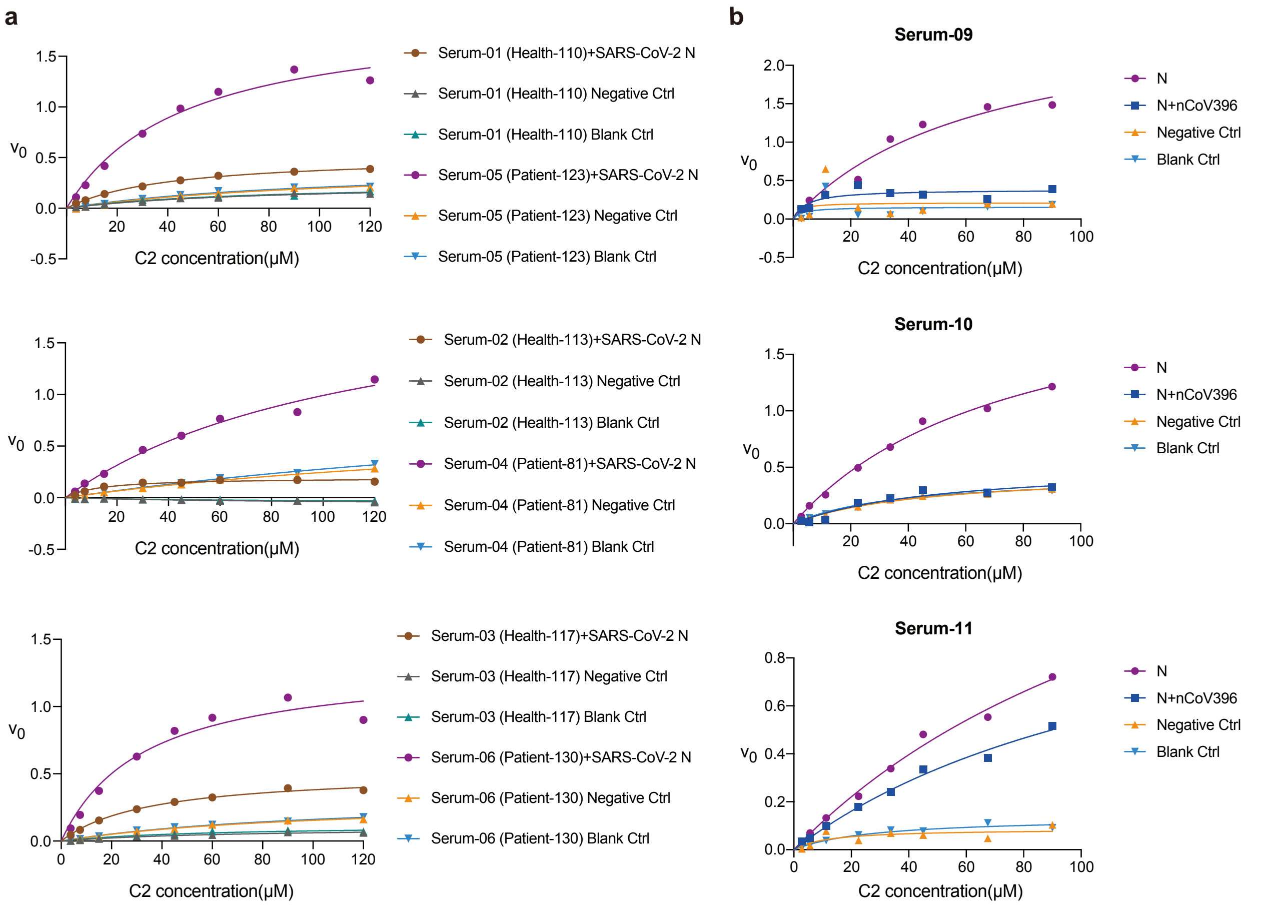

Supplementary Figure 5 | (a) Data are from three donors with abnormal serologic C3 value (patient) and three donors with normal serologic C3 and C4 values (health). The Michaelis-Menten curve shows the effect of the N protein in the former was higher than latter on the substrate C2 cleavage of MAPS-2. (**b**) The mAb nCoV396 inhibits the N protein-induced excessive cleavage of C2 in the serum with abnormal serologic C3 values. Negative Ctrl (orange curve) represents reactions containing another protein (ENL) expressed in *E coli*. instead of SARS-CoV-2 N protein, and Blank Ctrl (blue curve) without SARS-CoV-2 N protein. All samples were performed in triplicates and mean were presented.

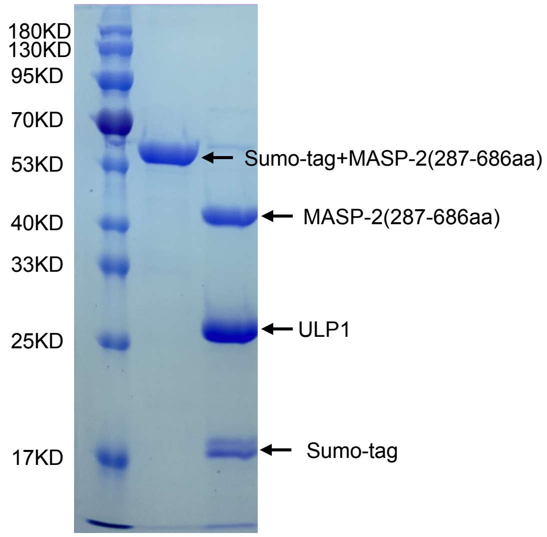

Supplementary Figure 6 | SDS-PAGE analysis of purified recombinant MASP-2 (287-686aa) fragments. Marker (left lane), SUMO-tag MASP-2 protein (middle lane, ranged 287-686 residues), and digested product of SUMO-tag MASP-2 protein with ULP1 protease are shown. The experiment was repeated three times independently with similar results.

a

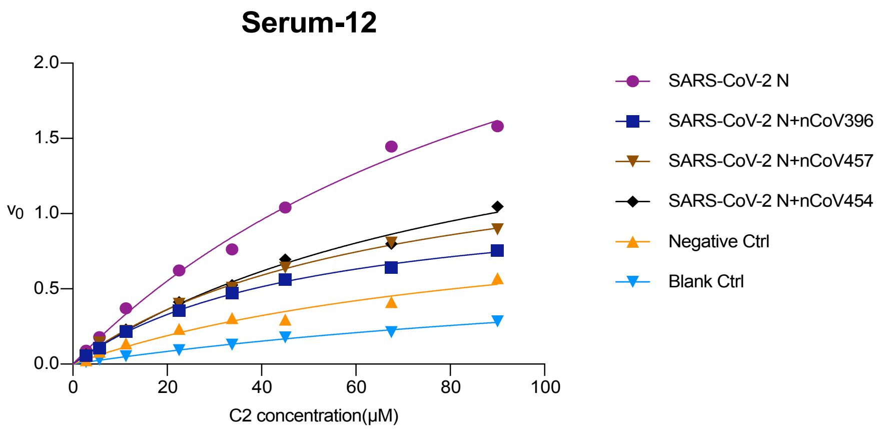

b

	SARS-CoV-2 N	SARS-CoV-2 N+nCoV396	SARS-CoV-2 N+nCoV457	SARS-CoV-2 N+nCoV454	Negative Ctrl	Blank Ctrl
Vmax (RU s-1) (95% CI)	3.612	1.165	1.574	2.06	1.098	0.8044
	(3.072-4.403)	(1.037-1.331)	(1.410-1.785)	(1.741-2.539)	(0.769-2.033)	(0.632-1.120)
Km(μM)	110.8	50.66	66.76	93.67	96.29	170.7
Vmax/Km(RU s-1/μM)	0.033	0.023	0.024	0.022	0.011	0.005

c

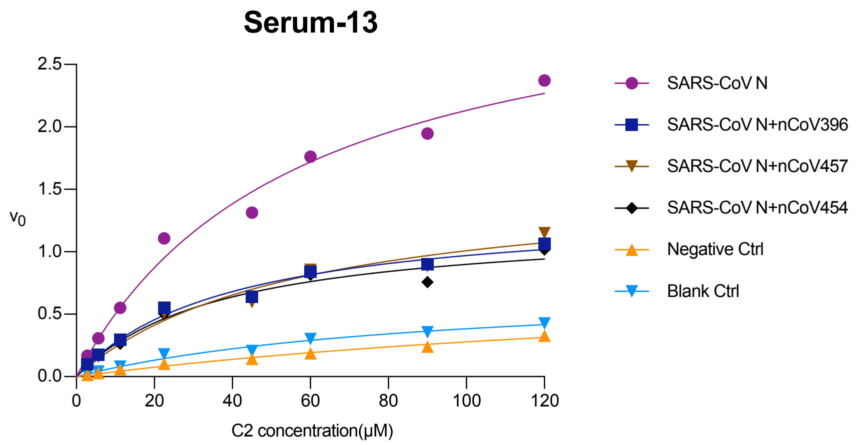

d

	SARS-CoV N	SARS-CoV N+nCoV396	SARS-CoV N+nCoV457	SARS-CoV N+nCoV454	Negative Ctrl	Blank Ctrl
Vmax (RU s-1) (95% CI)	3.335	1.352	1.579	1.236	0.8553	0.7359
	(2.978-3.805)	(1.239-1.491)	(1.307-2.025)	(1.034-1.553)	(0.597-1.584)	(0.615-0.923)
Km(μM)	56.42	39.42	56.59	37.44	207.4	91.98
Vmax/Km(RU s-1/μM)	0.059	0.034	0.028	0.033	0.004	0.008

Supplementary Figure 7 | Other antibodies (nCoV454 and nCoV457) compromise SARS-CoV-2 and SARS-CoV N-induced complement hyperactivation. (**a**) The Michealis-Menten curve of ex vivo SARS-CoV-2 N protein-induced excessive cleavage of C2 in the serum. The V_0 is calculated with and indicated on the Y-axis, the concentration of substrate is indicated on the X-axis. mAb nCoV396 (dark blue), nCoV457 (cyan), nCoV454 (black), Negative control with other protein expressed in *E. coli* (ENL) (orange), and Blank control (blue) are presenting. (**b**) The corresponding kinetics parameters of (**a**) are presented. (**c**) The Michealis-Menten curve of ex vivo SARS-CoV N protein-induced excessive cleavage of C2 in the serum, with similar representation as (**a**). (**d**) The corresponding kinetics parameters of (**c**) are presented. (**a**, **c**) All samples were performed in triplicates and mean were presented.

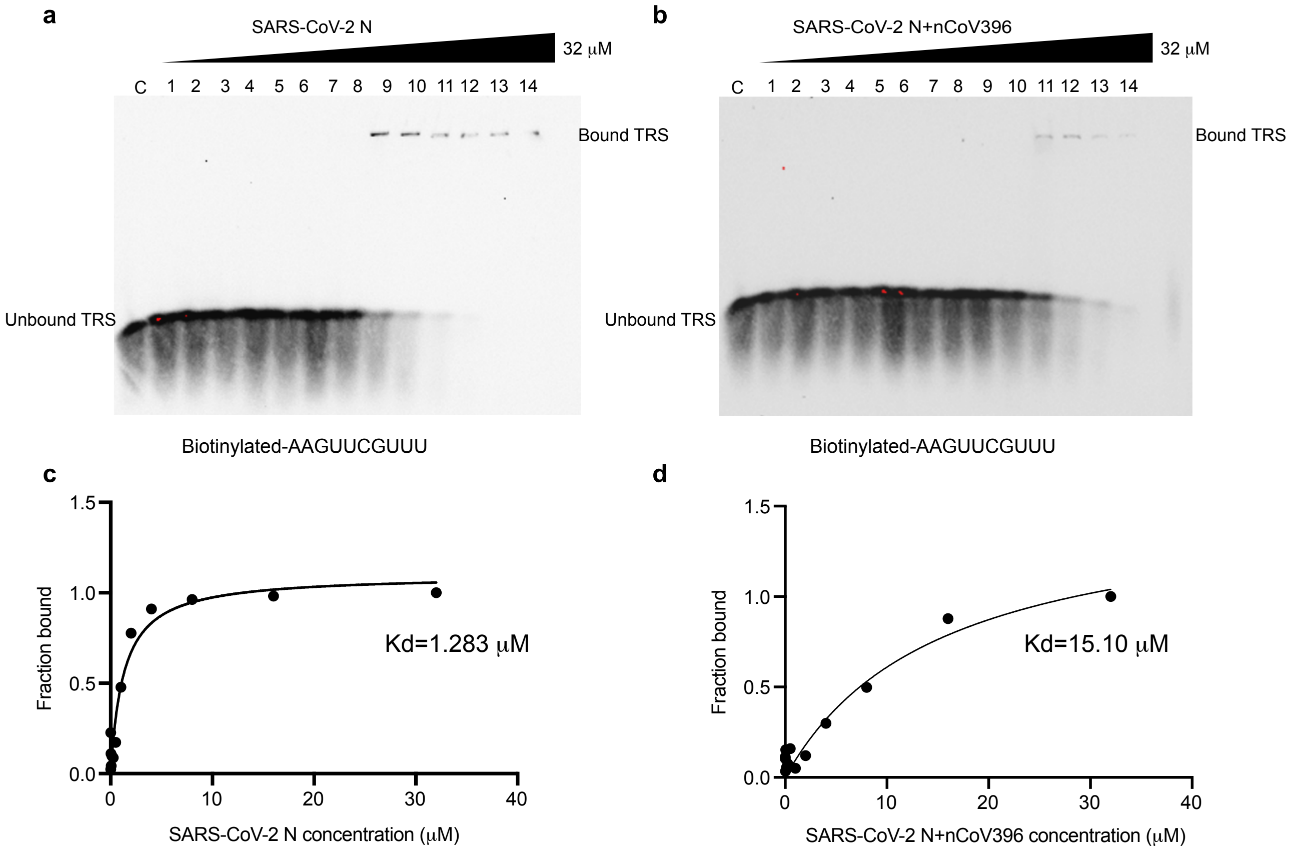

Supplementary Figure 8 | Electrophoretic mobility shift assays of SARS-CoV-2 N protein with viral TRS. Mobility shift of biotinylated TRS bound to SARS-CoV-2 N protein (**a**) and SARS-CoV-2 N protein mixed with mAb nCoV396 (Molar concentration ratio=1:1) (**b**). The protein concentration was increased by a factor of 2, starting from lane 1 (7.8125 nM) to lane 13 (32 μM). Lane C, negative control (only biotinylated TRS without protein). Lane 14, the highest concentration of protein without biotinylated TRS. (**c**) and (**d**) Fitting of the kinetic dissociation values of SARS-CoV-2 (**a**) and SARS-CoV-2 N protein mixed with mAb nCoV396 (**b**) based on the EMSA results. The SARS-CoV-2 N protein derive from the same experiment and EMSA gels（**a, b**） were processed in parallel.

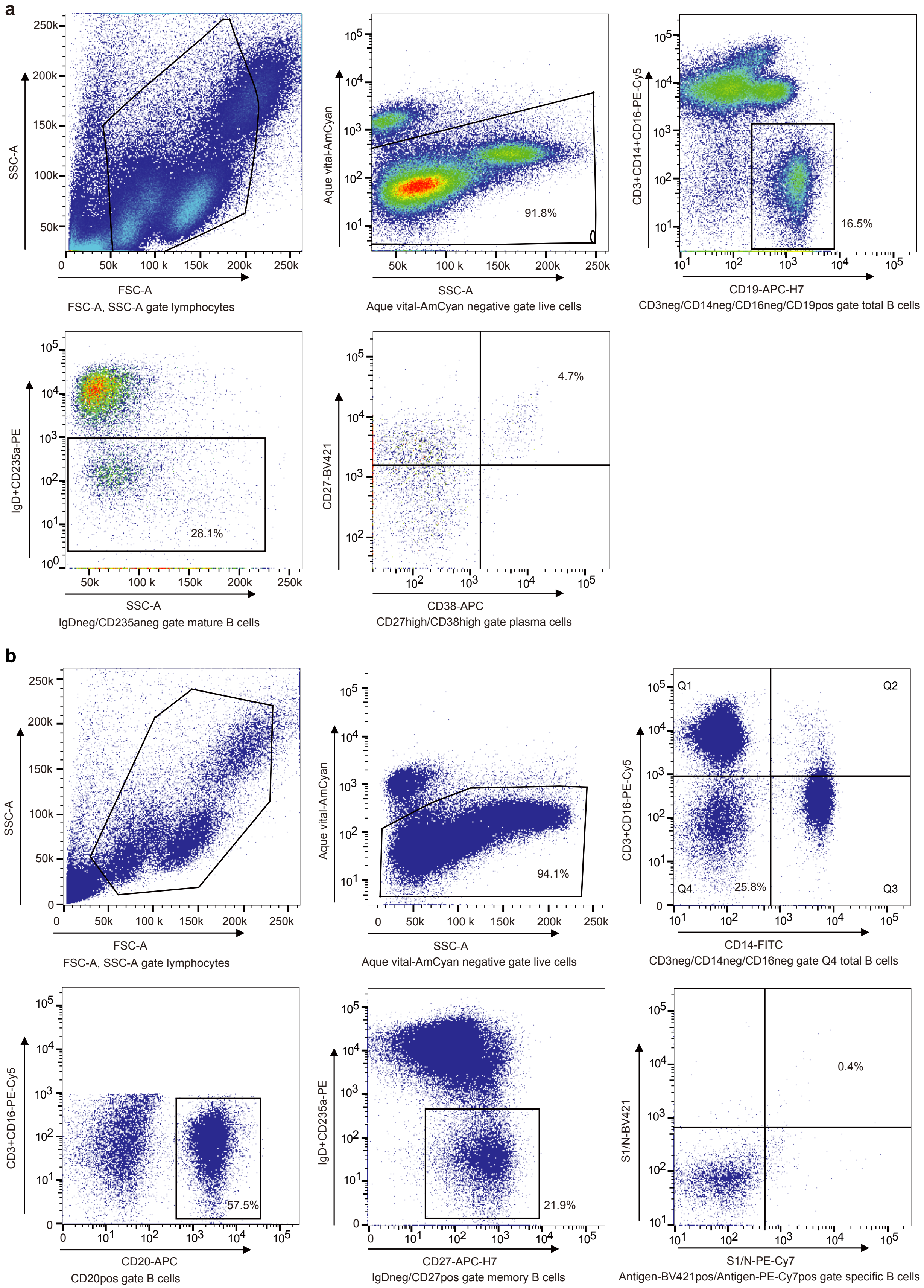

Supplementary Figure 9 | Gate strategy used for cell-surface staining analysis. (**a**) Gate strategy of Fig.1**c** for sorting single plasma cells with CD38 and CD27 double-positive B cells by FACS. (**b**) Gate strategy of Fig.1**d** for sorting antigen-specific memory B cells by FACS.

Supplementary Table 1. Clinical features of COVID-19 convalescents donors.

Patient ID	Days after disease onset	Serum antibody titers to SARS-CoV-2 S	Serum antibody titers to SARS-CoV-2 N
ZD002	19	1:36,450	1:36,450
ZD004	11	1:1,350	1:12,150
ZD005	23	1:36,450	1:36,450
ZD006	9	1:12,150	1:328,050
ZD007	17	1:450	1:109,350
ZD008	25	1:12,150	1:109,350

Supplementary Table 2. Ig gene family of collected N-protein monoclonal antibodies.

Antibody ID	V_H							$V_{\kappa/\lambda}$				
	IgH ID	V	D	J	Mutation frequency	CDR3 Length (No. a.a.)	Isotype	Igκ/λ ID	V	J	Mutation frequency	CDR3 Length (No. a.a.)
nCoV319	H16174	4-59	6-13	4	2.8%	10	IgG3	K12616	4-1	4	1.0%	9
nCoV344	H16239	3-7	3-9	4	0.4%	19	IgG1	K12665	3-20	5	0.7%	10
nCoV348	H16248	5-51	2-21	4	6.6%	20	IgG2	K12673	4-1	2	7.4%	9
nCoV365	H16299	5-51	3-10	6	8.7%	26	IgG2	K12707	4-1	4	7.1%	9
nCoV370	H16310	3-30	2-2	1	0.4%	24	IgG3	K12716	4-1	4	0.0%	9
nCoV377	H16334	4-59	3-9	4	0.0%	18	IgG1	K12729	3-20	1	0.0%	9
nCoV388	H16357	3-7	2-21	4	0.0%	20	IgG3	K12749	2-30	5	0.0%	20
nCoV390	H16365	4-59	3-10	1	0.0%	22	IgG3	K12753	3-20	3	0.0%	9
nCoV396	H16030	3-30	6-13	5	2.8%	14	IgG1	L5337	4-69	3	1.7%	9
nCoV400	H16042	3-23	6-13	6	0.0%	23	IgG3	L5344	3-25	3	0.0%	12
nCoV402	H16053	3-30	6-13	4	8.7%	14	IgG1	L5347	4-69	3	6.8%	9
nCoV405	H16064	3-30	6-13	4	7.6%	14	IgG1	L5353	4-69	2	7.5%	9
nCoV414	H16093	3-15	3-10	4	0.0%	18	IgA1	L5366	3-21	1	0.0%	11
nCoV416	H16097	1-46	4-23	4	11.1%	14	IgG1	L5369	4-69	3	6.1%	9
nCoV422	H16118	3-30	6-13	4	5.9%	14	IgG1	L5379	4-69	2	5.8%	9
nCoV424	H16127	1-46	3-16	4	8.0%	14	IgG1	L5381	4-69	3	4.4%	9
nCoV425	H16133	3-30	3-22	4	7.0%	14	IgA1	L5382	4-69	3	2.0%	9
nCoV431	H16168	1-8	5-24	4	9.7%	12	IgA1	L5389	3-25	3	7.2%	11
nCoV433	H16177	3-30	3-22	4	4.6%	14	IgA1	L5392	4-69	3	3.1%	9
nCoV439	H16214	1-2	3-16	4	0.0%	11	IgG1	L5401	1-40	2	0.7%	9
nCoV445	H16245	7-4	6-19	4	2.8%	12	IgG1	L5413	2-14	3	2.1%	10
nCoV454	H16287	3-30	6-13	4	8.1%	14	IgG1	L5427	4-69	3	3.7%	9
nCoV455	H16291	1-24	2-2	6	0.0%	22	IgA1	L5428	3-25	3	0.0%	11
nCoV457	H16304	3-30	5-24	4	8.7%	14	IgG1	L5431	4-69	3	5.1%	9
nCoV459	H16314	3-73	3-10	6	0.0%	17	IgG3	L5433	3-25	2	0.0%	11
nCoV463	H16327	3-64	4-17	4	3.8%	15	IgG1	L5437	2-14	3	0.7%	12
nCoV464	H16330	3-30	2-15	4	0.0%	15	IgG3	L5439	2-23	2	0.0%	10
nCoV468	H16339	3-30	4-17	4	0.4%	16	IgG3	L5446	2-23	3	0.0%	13
nCoV471	H16353	1-46	3-22	4	6.3%	17	IgG1	L5451	3-1	2	2.5%	9
nCoV472	H16359	3-30	6-13	4	0.7%	15	IgM	L5453	4-69	3	1.0%	9
nCoV474	H16363	3-33	4-23	5	6.9%	15	IgG1	L5456	3-10	3	3.2%	11
nCoV476	H16367	3-33	4-23	4	9.4%	15	IgA1	L5459	3-10	3	6.8%	11

Supplementary Table 3. SPR analysis of selected mAbs to nucleocapsid protein.

Ligand	Analyte	Ka (1/Ms)	Kd (1/s)	KD (M)
nCoV396	SARS-CoV-2 N protein	2833	2.89 E-06	1.02 E-09
nCoV416	SARS-CoV-2 N protein	5636	4.09 E-05	7.26 E-09
nCoV400	SARS-CoV-2 N protein	1.63 E+04	1.94 E-05	1.19 E-09
nCoV402	SARS-CoV-2 N protein	1.57 E+05	3.47 E-04	2.21 E-09
nCoV424	SARS-CoV-2 N protein	3.03 E+04	4.74 E-04	1.57 E-08
nCoV425	SARS-CoV-2 N protein	1.75 E+04	4.40 E-04	2.52 E-08
nCoV433	SARS-CoV-2 N protein	3.40 E+04	5.03 E-04	1.48 E-08
nCoV454	SARS-CoV-2 N protein	1.47 E+05	1.81 E-03	1.24 E-08
nCoV457	SARS-CoV-2 N protein	2.20 E+04	2.83 E-04	1.29 E-08
nCoV396	SARS-CoV N protein	3.97 E+04	2.95 E-04	7.44 E-09
nCoV396	MERS-CoV N protein	5.38 E+04	4.00 E-04	7.43 E-09

Supplementary Table 4. Data collection and refinement statistics.

	The Complex of mAb-396 with SARS-CoV-2 N-NTD (41-174)*
**Data collection**	SSRF BL-18U (PDB:7CR5)
Space group	$P\,2_1\,2_1\,2$
Cell dimensions	
a, *b*, *c* (Å)	154.07, 52.60, 85.30
α, β, γ (°)	90, 90, 90
Resolution (Å)	50 - 2.1 (2.14 - 2.1) **
R_{merge}#	0.22 (1.33)
$I / \sigma(I)$	16.07 (1.64)
Completeness (%)	99.5 (93.7)
Redundancy	12.1 (8.4)
**Refinement**	
Resolution (Å)	28.43 - 2.1 (2.16 - 2.1)
No. reflections	41691 (3561)
R_{work}/ R_{free} (%) ##	0.19 / 0.22
No. atoms	4434
Protein	4178
Ligand/ion	1
Water	255
B-factors (Å^2)	35.39
Protein	35.19
Ligand/ion	73.44
Water	38.54
R.m.s. deviations	
Bond lengths (Å)	0.007
Bond angles (°)	0.87
Ramachandran plot (%)	
Favored	97.1
Allowed	2.9
Disallowed	0.0

*This dataset is collected with one crystal.

**Values in parentheses are for the highest-resolution shell.

#Rmerge = ∑ hkl ∑ i |Ii (hkl)- < I(hkl) >| / ∑ hkl ∑ i Ii(hkl), where Ii(hkl) is the intensity measured for the is the reflection and < I(hkl) > is the average intensity of all reflections with indices hkl.

##R-work = ∑ hkl ||Fobs (hkl) |-|Fcalc (hkl) || / ∑ hkl |Fobs(hkl) |. R-free is calculated in an identical manner using 10% of randomly selected reflections that were not included in the refinement.

Supplementary Table 5. PDBePISA Interfaces Calculation to the complex of N-NTD with mAb-396.

Structure 1	Structure 2	Interface area, Å^{2} (a)	$\Delta^{i}G$ kcal/mol (b)	$\Delta^{i}G$ P-value(c)	N_{HB}(d)	N_{SB}(e)	N_{DS}(f)	CSS(g)
Light Chain	Heavy Chain	1456.8	-21.0	0.095	16	3	0	1.000
Light Chain	N-NTD	659.8	-2.9	0.583	13	0	0	0.213
Heavy Chain	N-NTD	420.9	-5.7	0.217	5	1	0	0.104
Light Chain	Light Chain	516.4	-3.8	0.441	4	0	0	0.000
Heavy Chain	Heavy Chain	19.2	-0.5	0.365	0	0	0	0.000
N-NTD	N-NTD	344.9	-3.7	0.273	2	0	0	0.000

a Interface area in Å^{2}, calculated as difference in total accessible surface areas of isolated and interfacing structures divided by two;

b $\Delta^{i}G$ indicates the solvation free energy gain upon formation of the interface, in kcal/M. The value is calculated as difference in total solvation energies of isolated and interfacing structures;

c $\Delta^{i}G$ P-value indicates the P-value of the observed solvation free energy gain;

d N_{HB} indicates the number of potential hydrogen bonds across the interface;

e N_{SB} indicates the number of potential salt bridges across the interface;

f N_{DS} indicates the number of potential disulfide bonds across the interface;

g CSS stands for the Complexation Significance Score, which indicates how significant for assembly formation the interface is.

Supplementary Table 6. Clinical features of donors with serologic C3 values.

Sample ID	Patient ID	C3 (0.7-1.4 g/L)[(a)]	C4 (0.1-0.4 g/L)[(a)]	Application
Serum-01	Health-110	1.00	0.22	Fig. 4b<br>Supplementary Fig. 5a
Serum-02	Health-113	0.84	0.25	Fig. 4b<br>Supplementary Fig. 5a
Serum-03	Health-117	1.14	0.33	Fig. 4b<br>Supplementary Fig. 5a
Serum-04	Patient-81	1.48	0.21	Fig. 4b<br>Supplementary Fig. 5a
Serum-05	Patient-123	1.55	0.26	Fig. 4b<br>Supplementary Fig. 5a
Serum-06	Patient-130	1.66	0.23	Fig. 4b<br>Supplementary Fig. 5a
Serum-07	Patient-49	1.43	0.26	Fig. 4d
Serum-08	Patient-20	1.57	0.37	Fig. 4f, g
Serum-09	Patient-19	1.47	0.23	Fig. 4g<br>Supplementary Fig. 5b
Serum-10	Patient-34	1.43	0.37	Fig. 4g<br>Supplementary Fig. 5b
Serum-11	Patient-38	1.57	0.28	Fig. 4g<br>Supplementary Fig. 5b
Serum-12	Patient-71	1.58	0.31	Fig. 4g,<br>Supplementary Fig. 7a
Serum-13	Patient-72	1.48	0.28	Supplementary Fig. 7c

a Reference value range for healthy person. All serum samples were detected by Clinical Laboratory Group of Department of Experimental Medicine of The Fifth Affiliated Hospital, Sun Yat-sen University.

Supplementary Table 7. The kinetic enzyme parameters of viral protein-induced complement activation analysis in donors with abnormal and normal serologic C3 values (Fig. 4b).

Sample ID	Groups	Vmax (95% CI) (RU s^{-1})	Km (μM)
Serum-01	N	0.5228(0.4870-0.5645)	40.42
	Negative Ctrl	0.2557(0.2068-0.3376)	79.97
	Blank Ctrl	0.2524(0.2113-0.3160)	71.13
Serum-02	N	0.1944(0.1794-0.2114)	13.80
	Negative Ctrl	-0.0699(-0.6590—0.0389)	92.95
	Blank Ctrl	-0.04826(-6.663—0.02537)	69.69
Serum-03	N	0.5150 (0.4859-0.5480)	35.18
	Negative Ctrl	0.1042(0.07338-0.1862)	70.13
	Blank Ctrl	0.1188(0.0931-0.1648)	57.09
Serum-04	N	2.150(1.720-2.905)	117.0
	Negative Ctrl	0.8044(0.6322-1.120)	227.7
	Blank Ctrl	1.189(0.8049-2.387)	327.9
Serum-05	N	1.922(1.675-2.257)	46.29
	Negative Ctrl	0.4285(0.2923-0.8524)	116.0
	Blank Ctrl	0.4236(0.3471-0.5478)	103.9
Serum-06	N	1.327(1.158-1.551)	32.94
	Negative Ctrl	0.3146(0.2346-0.4886)	104.2
	Blank Ctrl	0.3324(0.2667-0.4471)	106.8

a. Changes in Vmax are obtained by divided by the values of Blank Ctrl in each patient.

Supplementary Table 8. Comparison of kinetics of cleavage of synthetic peptide substrates representing physiological cleavage sequences by recombinant MASP-2 (287-686aa) fragment without and with different concentration SARS-CoV-2 N protein (Fig. 4c).

	MASP-2	MASP-2+SARS-CoV-2 N(1:12.5)	MASP-2+SARS-CoV-2 N(1:25)	MASP-2+SARS-CoV-2 N(1:50)	Negative Ctrl
Vmax (RU s-1)	1.75	1.164	1.471	2.024	1.259
(95% CI)	(0.898-35.60)	(0.941-1.541)	(1.250-1.795)	(1.819-2.283)	(1.037-1.614)
Km(μM)	156.7	54.37	34.88	24.11	108.1
Kcat(s-1)	5.469	3.638	4.597	6.325	3.378
Kcat/Km	0.035	0.067	0.132	0.262	0.031

Supplementary Table 9. The kinetic enzyme parameters of viral protein-induced complement hyper-activation analysis in serum-07 (Fig. 4d).

	Michaelis-Menten		Allosteric sigmoidal			
	**Vmax (RU·s-1)**	**Km (μM)**	**Vmax (RU·s-1)**	**h**	**$K_{0.5}$ (μM)**	**Kprime**
N(10μM)	2.748	63.54	2.178	1.219	40.07	89.89
N(7.5μM)	2.596	55.11	2.191	1.164	38.93	70.88
N(5μM)	3.021	68.59	2.429	1.186	44.61	90.51
N(2.5μM)	3.002	67.64	2.353	1.221	41.91	95.84
N(0.5μM)	2.376	57.31	1.811	1.309	33.51	99.11
Negative Ctrl	1.417	51.3	1.146	1.237	33.33	76.57
Blank Ctrl	1.492	59.63	1.281	1.132	43.71	72.03

Supplementary Table 10. The kinetic enzyme parameters of viral protein-induced complement hyper-activation analysis in serum-08 (Fig. 4f).

	**Michaelis-Menten**		**Allosteric sigmoidal**			
	**Vmax (RU·s-1)**	**Km (μM)**	**Vmax (RU·s-1)**	**h**	**$K_{0.5}$ (μM)**	**Kprime**
N	3.855	162.900	2.105	1.355	58.700	249.400
N+nCoV396(1:0.25)	1.996	137.000	1.195	1.343	56.130	223.500
N+nCoV396(1:0.5)	1.161	222.600	0.441	1.590	48.000	471.600
N+nCoV396(1:1)	0.064	1.631	0.112	/[(a)]	0.029	1.000
N+nCoV396(1:1.5)	0.019	27.460	0.026	133.3	30.480	/
N+nCoV396(1:2)	0.036	2.261	0.061	/	/	1.000
Negative Ctrl	0.011	3.424	0.017	/	0.198	1.000
Blank Ctrl	0.041	7.274	0.038	1.679	7.474	29.31

a. The slash symbol indicates the value is ambiguous since the Allosteric sigmoidal cannot be fit well.

Supplementary Table 11. The kinetic enzyme parameters of viral protein-induced complement hyper-activation analysis (Fig. 4d, f, supplementary fig. 5b, 7a).

Sample ID	Groups	Vmax (95% CI) (RU s^{-1})	Changes in Vmax[(a)]	Km (μM)	Vmax/Km (RU s^{-1}/μM)
Serum-07	N(10 μM)	2.748(2.573-2.950)	1.842	63.540	0.043
	N(7.5 μM)	2.596(2.468-2.740)	1.740	55.110	0.047
	N(5 μM)	3.021(2.780-3.311)	2.025	68.590	0.044
	N(2.5 μM)	3.002(2.724-3.349)	2.012	67.640	0.044
	N(0.5 μM)	2.376(2.192-2.595)	1.592	57.310	0.041
	Negative Ctrl	1.417(1.315-1.537)	0.950	51.300	0.028
	Blank Ctrl	1.492(1.405-1.590)	1	59.630	0.025
Serum-08	N	3.855(2.850-6.094)	94.393	162.900	0.024
	N+nCoV396(1:0.25)	1.996(1.413-3.511)	48.874	137.000	0.015
	N+nCoV396(1:0.5)	1.161(ND)[(b)]	28.428	222.600	0.005
	N+nCoV396(1:1)	0.064(0.016-0.138)	1.560	1.631	0.039
	N+nCoV396(1:1.5)	0.019(ND)	0.473	27.460	0.001
	N+nCoV396(1:2)	0.036(ND)	0.881	2.261	0.016
	Negative Ctrl	0.011(ND)	0.266	3.424	0.003
	Blank Ctrl	0.041(ND)	1	7.274	-0.006
Serum-09	N	2.745(1.827-5.819)	17.540	65.550	0.042
	N+nCoV396	0.383(0.316-0.466)	2.449	4.705	0.081
	Negative Ctrl	0.212(0.105-0.357)	1.355	1.902	0.111
	Blank Ctrl	0.157(0.077-0.369)	1	3.018	0.052
Serum-10	N	2.257(2.016-2.569)	5.203	76.950	0.029
	N+nCoV396	0.529(0.386-0.861)	1.219	51.510	0.010
	Negative Ctrl	0.467(0.408-0.549)	1.077	46.050	0.010
	Blank Ctrl	0.434(0.382-0.503)	1	36.380	0.012
Serum-11	N	1.956(1.566-2.634)	14.330	157.500	0.012
	N+nCoV396	1.260(1.018-1.671)	9.231	136.800	0.009
	Negative Ctrl	0.087(0.054-0.285)	0.635	12.010	0.007
	Blank Ctrl	0.137(0.105-0.195)	1	28.030	0.005
Serum-12	N	3.612(3.072-4.403)	4.493	110.800	0.043
	N+nCoV396	1.165(1.037-1.331)	1.449	50.660	0.047
	Negative Ctrl	1.098(0.769-2.033)	1.366	96.290	0.044
	Blank Ctrl	0.804(0.632-1.120)	1	170.700	-0.006

a. Changes in Vmax are obtained by divided by the values of Blank Ctrl in each patient.

b. ND indicates the value is not determined since the Michaelis-Menten kinetics cannot be fit well.

Supplementary Table 12. SARS-CoV-2 N protein antibodies epitope ELISA analysis.

Peptide Number	Peptide sequence (N→C)	nCoV396			nCoV454			nCoV457			nCoV416			TT0170		
NP1(1-18aa)	MSDNGPQNQRNAPRITFGGSK(Biotin)	0.021	0.016	0.013	0.022	0.022	0.029	0.049	0.038	0.035	0.008	0.013	0.013	0.011	0.011	0.015
NP2(7-24aa)	QNQRNAPRITFGGPSDSTGSK(Biotin)	0.014	0.009	0.005	0.016	0.016	0.027	0.039	0.029	0.026	0.004	0.005	0.01	0.004	0.004	0.012
NP3(13-30aa)	PRITFGGPSDSTGSNQNGGSK(Biotin)	0.016	0.009	0.007	0.018	0.022	0.031	0.041	0.032	0.028	0.005	0.008	0.01	0.006	0.008	0.015
NP4(19-36aa)	GPSDSTGSNQNGERSGARGSK(Biotin)	0.017	0.011	0.007	0.022	0.023	0.032	0.041	0.032	0.028	0.006	0.009	0.016	0.008	0.011	0.017
NP5(25-42ss)	GSNQNGERSGARSKQRRPGSK(Biotin)	0.019	0.013	0.008	0.023	0.022	0.029	0.037	0.03	0.028	0.007	0.01	0.015	0.009	0.012	0.015
NP6(31-48aa)	ERSGARSKQRRPQGLPNNGSK(Biotin)	0.018	0.012	0.005	0.022	0.023	0.037	0.041	0.03	0.025	0.007	0.012	0.014	0.008	0.01	0.014
NP7(37-54aa)	SKQRRPQGLPNNTASWFTGSK(Biotin)	0.028	0.015	0.017	0.03	0.027	0.032	0.042	0.031	0.03	0.015	0.015	0.02	0.012	0.01	0.017
NP8(43-60aa)	QGLPNNTASWFTALTQHGGSK(Biotin)	0.021	0.013	0.015	0.028	0.03	0.037	0.026	0.024	0.022	0.01	0.012	0.021	0.008	0.007	0.011
NP9(49-66aa)	TASWFTALTQHGKEDLKFGSK(Biotin)	0.023	0.017	0.016	0.027	0.039	0.038	0.034	0.026	0.023	0.01	0.013	0.02	0.007	0.012	0.014
NP10(55-72aa)	ALTQHGKEDLKFPRGQGVGSK(Biotin)	0.027	0.016	0.017	0.028	0.036	0.047	0.039	0.031	0.024	0.01	0.012	0.02	0.007	0.012	0.015
NP11(61-78aa)	KEDLKFPRGQGVPINTNSGSK(Biotin)	0.025	0.016	0.016	0.031	0.033	0.045	0.032	0.025	0.022	0.01	0.013	0.018	0.007	0.008	0.015
NP12(67-84aa)	PRGQGVPINTNSSPDDQIGSK(Biotin)	0.024	0.016	0.015	0.031	0.036	0.046	0.041	0.026	0.023	0.01	0.011	0.019	0.007	0.006	0.014
NP13(73-90aa)	PINTNSSPDDQIGYYRRAGSK(Biotin)	0.029	0.025	0.025	0.03	0.033	0.033	0.066	0.06	0.056	0.022	0.023	0.021	0.036	0.03	0.028
NP14(79-96aa)	SPDDQIGYYRRATRRIRGGSK(Biotin)	0.025	0.025	0.026	0.02	0.024	0.027	0.061	0.044	0.038	0.017	0.017	0.024	0.023	0.026	0.032
NP15(85-102aa)	GYYRRATRRIRGGDGKMKGSK(Biotin)	0.03	0.028	0.023	0.023	0.025	0.028	0.067	0.051	0.047	0.019	0.025	0.023	0.029	0.035	0.037
NP16(91-108aa)	TRRIRGGDGKMKDLSPRWGSK(Biotin)	0.033	0.03	0.027	0.025	0.026	0.029	0.059	0.053	0.056	0.024	0.022	0.026	0.034	0.035	0.038
NP17(97-114aa)	GDGKMKDLSPRWYFYYLGGSK(Biotin)	0.026	0.034	0.031	0.024	0.028	0.031	0.065	0.056	0.061	0.02	0.02	0.03	0.04	0.034	0.044
NP18(103-120aa)	DLSPRWYFYYLGTGPEAGGSK(Biotin)	0.031	0.027	0.03	0.026	0.027	0.03	0.062	0.056	0.063	0.013	0.014	0.027	0.031	0.032	0.041
NP19(109-126aa)	YFYYLGTGPEAGLPYGANGSK(Biotin)	0.035	0.024	0.024	0.032	0.026	0.033	0.044	0.037	0.044	0.019	0.016	0.02	0.016	0.015	0.042
NP20(115-132aa)	TGPEAGLPYGANKDGIIWGSK(Biotin)	0.021	0.015	0.012	0.028	0.025	0.036	0.045	0.031	0.026	0.007	0.008	0.018	0.013	0.008	0.03
NP21(121-138aa)	LPYGANKDGIIWVATEGAGSK(Biotin)	0.027	0.018	0.014	0.029	0.023	0.036	0.04	0.03	0.031	0.01	0.011	0.013	0.012	0.009	0.027
NP22(127-144aa)	KDGIIWVATEGALNTPKDGSK(Biotin)	0.026	0.018	0.012	0.031	0.029	0.046	0.044	0.03	0.032	0.011	0.011	0.02	0.013	0.013	0.032
NP23(133-150aa)	VATEGALNTPKDHIGTRNGSK(Biotin)	0.022	0.016	0.011	0.033	0.031	0.041	0.044	0.032	0.036	0.01	0.01	0.017	0.013	0.012	0.034
NP24(139-156aa)	LNTPKDHIGTRNPANNAAGSK(Biotin)	0.021	0.011	0.01	0.031	0.033	0.049	0.047	0.039	0.033	0.01	0.01	0.018	0.013	0.014	0.031
NP25(145-162aa)	HIGTRNPANNAAIVLQLPGSK(Biotin)	0.031	0.027	0.026	0.052	0.044	0.054	0.036	0.034	0.033	0.023	0.023	0.035	0.011	0.017	0.023
NP26(151-168aa)	PANNAAIVLQLPQGTTLPGSK(Biotin)	0.02	0.023	0.022	0.041	0.04	0.054	0.041	0.031	0.028	0.019	0.016	0.026	0.007	0.01	0.015
NP27(157-174aa)	IVLQLPQGTTLPKGFYAEGSK(Biotin)	2.439	2.02	2.34	1.695	1.79	2.03	2.193	2.142	1.648	2.171	2.381	2.641	0.007	0.011	0.018
NP28(163-180aa)	QGTTLPKGFYAEGSRGGSGSK(Biotin)	2.215	2.007	1.83	1.86	1.844	2.216	2.047	1.795	1.601	1.719	1.86	2.12	0.009	0.014	0.02
NP29(169-186aa)	KGFYAEGSRGGSQASSRSGSK(Biotin)	0.03	0.023	0.028	0.043	0.042	0.059	0.034	0.031	0.023	0.018	0.018	0.033	0.011	0.018	0.02
NP30(175-192aa)	GSRGGSQASSRSSSRSRNGSK(Biotin)	0.028	0.021	0.023	0.04	0.038	0.069	0.037	0.029	0.022	0.02	0.016	0.033	0.008	0.012	0.018
NP31(181-198aa)	QASSRSSSRSRNSSRNSTGSK(Biotin)	0.018	0.022	0.027	0.034	0.031	0.042	0.045	0.045	0.046	0.015	0.017	0.018	0.037	0.032	0.041
NP32(187-204aa)	SSRSRNSSRNSTPGSSRGGSK(Biotin)	0.018	0.03	0.024	0.023	0.026	0.042	0.047	0.04	0.042	0.009	0.016	0.023	0.024	0.023	0.035
NP33(193-210aa)	SSRNSTPGSSRGTSPARMGSK(Biotin)	0.021	0.028	0.023	0.024	0.027	0.043	0.051	0.048	0.049	0.015	0.018	0.021	0.028	0.024	0.036
NP34(199-216aa)	PGSSRGTSPARMAGNGGDGSK(Biotin)	0.027	0.023	0.024	0.026	0.027	0.043	0.054	0.052	0.058	0.017	0.023	0.025	0.033	0.029	0.047
NP35(205-222aa)	TSPARMAGNGGDAALALLGSK(Biotin)	0.026	0.03	0.029	0.029	0.03	0.044	0.054	0.048	0.053	0.025	0.021	0.025	0.035	0.029	0.045
NP36(211-228aa)	AGNGGDAALALLLLDRLNGSK(Biotin)	0.02	0.031	0.031	0.025	0.032	0.039	0.051	0.048	0.053	0.023	0.021	0.025	0.041	0.03	0.045
NP37(217-234aa)	AALALLLLDRLNQLESKMGSK(Biotin)	0.015	0.013	0.015	0.032	0.03	0.043	0.045	0.034	0.029	0.01	0.009	0.012	0.01	0.012	0.015
NP38(223-240aa)	LLDRLNQLESKMSGKGQQGSK(Biotin)	0.012	0.012	0.01	0.026	0.025	0.039	0.036	0.031	0.024	0.005	0.006	0.009	0.006	0.005	0.012
NP39(229-246aa)	QLESKMSGKGQQQQGQTVGSK(Biotin)	0.019	0.013	0.012	0.026	0.029	0.035	0.038	0.032	0.028	0.006	0.008	0.011	0.011	0.011	0.016
NP40(235-252aa)	SGKGQQQQGQTVTKKSAAGSK(Biotin)	0.019	0.014	0.013	0.032	0.034	0.041	0.041	0.034	0.028	0.008	0.008	0.012	0.013	0.013	0.017
NP41(241-258aa)	QQGQTVTKKSAAEASKKPGSK(Biotin)	0.024	0.016	0.013	0.033	0.035	0.044	0.041	0.036	0.028	0.008	0.009	0.013	0.012	0.011	0.015
NP42(247-264aa)	TKKSAAEASKKPRQKRTAGSK(Biotin)	0.017	0.013	0.012	0.028	0.029	0.033	0.045	0.036	0.026	0.008	0.01	0.014	0.01	0.01	0.013
NP43(253-270aa)	EASKKPRQKRTATKAYNVGSK(Biotin)	0.02	0.016	0.013	0.038	0.038	0.041	0.056	0.044	0.048	0.012	0.008	0.023	0.023	0.022	0.029
NP44(259-276aa)	RQKRTATKAYNVTQAFGRGSK(Biotin)	0.019	0.01	0.007	0.03	0.023	0.044	0.062	0.036	0.032	0.005	0.005	0.019	0.012	0.012	0.025
NP45(265-282aa)	TKAYNVTQAFGRRGPEQTGSK(Biotin)	0.022	0.012	0.008	0.035	0.033	0.041	0.048	0.039	0.034	0.005	0.009	0.023	0.01	0.011	0.022
NP46(271-288aa)	TQAFGRRGPEQTQGNFGDGSK(Biotin)	0.022	0.009	0.01	0.035	0.036	0.035	0.052	0.039	0.034	0.007	0.009	0.025	0.011	0.011	0.022
NP47(277-294aa)	RGPEQTQGNFGDQELIRQGSK(Biotin)	0.018	0.013	0.01	0.044	0.038	0.04	0.05	0.039	0.035	0.009	0.01	0.029	0.013	0.012	0.022
NP48(283-300aa)	QGNFGDQELIRQGTDYKHGSK(Biotin)	0.016	0.012	0.009	0.041	0.035	0.039	0.058	0.04	0.037	0.007	0.009	0.028	0.015	0.014	0.024
NP49(289-306aa)	QELIRQGTDYKHWPQIAQGSK(Biotin)	0.016	0.012	0.017	0.023	0.028	0.037	0.061	0.048	0.041	0.009	0.012	0.013	0.021	0.026	0.02
NP50(295-312aa)	GTDYKHWPQIAQFAPSASGSK(Biotin)	0.015	0.009	0.012	0.023	0.024	0.039	0.062	0.038	0.033	0.014	0.008	0.013	0.021	0.018	0.024
NP51(301-318aa)	WPQIAQFAPSASAFFGMSGSK(Biotin)	0.015	0.014	0.026	0.024	0.025	0.039	0.066	0.043	0.061	0.013	0.014	0.015	0.025	0.027	0.031
NP52(301-324aa)	FAPSASAFFGMSRIGMEVGSK(Biotin)	0.015	0.02	0.022	0.027	0.029	0.041	0.061	0.045	0.054	0.015	0.011	0.014	0.031	0.027	0.024
NP53(313-330aa)	AFFGMSRIGMEVTPSGTWGSK(Biotin)	0.017	0.016	0.023	0.025	0.027	0.031	0.06	0.04	0.051	0.015	0.014	0.014	0.029	0.023	0.027
NP54(319-336aa)	RIGMEVTPSGTWLTYTAAGSK(Biotin)	0.016	0.014	0.017	0.024	0.026	0.035	0.048	0.049	0.044	0.013	0.014	0.015	0.03	0.024	0.036
NP55(325-343aa)	TPSGTWLTYTAAIKLDDKGSK(Biotin)	0.037	0.022	0.023	0.033	0.028	0.033	0.042	0.04	0.04	0.01	0.009	0.017	0.023	0.018	0.023
NP56(331-348aa)	LTYTAAIKLDDKDPNFKDGSK(Biotin)	0.03	0.012	0.011	0.028	0.023	0.036	0.038	0.036	0.032	-0.003	-1E-03	0.011	0.019	0.016	0.03
NP57(337-354aa)	IKLDDKDPNFKDQVILLNGSK(Biotin)	0.033	0.017	0.01	0.028	0.028	0.036	0.045	0.042	0.035	-0.005	-0.002	0.008	0.019	0.018	0.03
NP58(343-360aa)	DPNFKDQVILLNKHIDAYGSK(Biotin)	0.031	0.017	0.012	0.032	0.029	0.039	0.047	0.043	0.036	-0.002	0.001	0.011	0.021	0.019	0.032
NP59(349-366aa)	QVILLNKHIDAYKTFPPTGSK(Biotin)	0.03	0.013	0.009	0.034	0.031	0.04	0.043	0.039	0.04	0.002	0.004	0.013	0.021	0.02	0.035
NP60(355-372aa)	KHIDAYKTFPPTEPKKDKGSK(Biotin)	0.032	0.01	0.006	0.033	0.03	0.04	0.04	0.04	0.038	0.002	0.003	0.014	0.021	0.02	0.036
NP61(361-378aa)	KTFPPTEPKKDKKKKADEGSK(Biotin)	0.034	0.021	0.022	0.041	0.038	0.044	0.038	0.032	0.034	0.013	0.015	0.021	0.011	0.016	0.023
NP62(367-384aa)	EPKKDKKKKADETQALPQGSK(Biotin)	0.024	0.015	0.013	0.034	0.038	0.041	0.04	0.027	0.027	0.011	0.01	0.014	0.008	0.007	0.018
NP63(373-390aa)	KKKADETQALPQRQKKQQGSK(Biotin)	0.02	0.015	0.014	0.039	0.04	0.053	0.035	0.023	0.025	0.012	0.013	0.016	0.004	0.007	0.021
NP64(379-396aa)	TQALPQRQKKQQTVTLLPGSK(Biotin)	0.021	0.015	0.013	0.041	0.042	0.053	0.037	0.024	0.022	0.006	0.008	0.018	0.008	0.006	0.018
NP65(385-402aa)	RQKKQQTVTLLPAADLDDGSK(Biotin)	0.026	0.018	0.014	0.036	0.037	0.047	0.036	0.023	0.021	0.006	0.008	0.011	0.008	0.008	0.018
NP66(391-408aa)	TVTLLPAADLDDFSKQLQGSK(Biotin)	0.02	0.015	0.011	0.038	0.043	0.06	0.045	0.026	0.025	0.004	0.005	0.01	0.007	0.009	0.021
NP67(397-414aa)	AADLDDFSKQLQQSMSSAGSK(Biotin)	0.02	0.014	0.009	0.032	0.026	0.04	0.046	0.031	0.029	0.008	0.009	0.021	0.01	0.01	0.012
NP68(403-419aa)	FSKQLQQSMSSADSTQAAGSK(Biotin)	0.016	0.01	0.007	0.027	0.028	0.031	0.039	0.03	0.029	0.009	0.005	0.019	0.007	0.007	0.011

Supplementary Table 13. Oligonucleotides used for molecular cloning and EMSA.

Name	Sequence (5'-3')
SARS-CoV-2 N-NTD F	CGCGGATCCCGTCCGCAAGGTCTGCCGAA
SARS-CoV-2 N-NTD R	CCGCTCGAGTTACTCCGCATAGAAACCTTTCGG
SARS-CoV-2 N-CTD F	CGCGGATCCAGCGCGGCGGAAGCGAGCAA
SARS-CoV-2 N-CTD R	CCGCTCGAGTTACGGAAAGGTTTTGTACGCGTCG
SARS-CoV N-FL F	CGCGGATCCATGTCTGACAACGGTC
SARS-CoV N-FL R	CCGCTCGAGTTAAGCCTGGGTAGAGTCAGCAGAA
MERS-CoV N-FL F	CGCGGATCCATGGCTTCTCCGGCT
MERS-CoV N-FL R	CGCTCGAGTTAGTCGGTGTTAACGTCGATC
MASP-2 F	CGCGGATCCACCGGTTGGAAAATCCACTACA
MASP-2 R	CCGCTCGAGTTAGAAGTCAGAGATGATGTTTTCGA
TRS	Biotinylated-AAGUUCGUUU
