# Supplementary material for: A SARS-CoV-2 antibody curbs viral nucleocapsid protein-induced complement hyperactivation
Source: Nat Commun. 2021 May 11;12:2697. doi: 10.1038/s41467-021-23036-9 (PMC8113585; doi:10.1038/s41467-021-23036-9)
Supplement: Supplementary file 3 — Reporting Summary [file 41467_2021_23036_MOESM3_ESM.pdf]

nature research

Corresponding author(s): Shoudeng Chen

Last updated by author(s): Mar 18, 2021

Reporting Summary

Nature Research wishes to improve the reproducibility of the work that we publish. This form provides structure for consistency and transparency in reporting. For further information on Nature Research policies, see our Editorial Policies and the Editorial Policy Checklist.

Statistics

For all statistical analyses, confirm that the following items are present in the figure legend, table legend, main text, or Methods section.

n/a	Confirmed	
☐	☒	The exact sample size (*n*) for each experimental group/condition, given as a discrete number and unit of measurement
☐	☒	A statement on whether measurements were taken from distinct samples or whether the same sample was measured repeatedly
☐	☒	The statistical test(s) used AND whether they are one- or two-sided<br>*Only common tests should be described solely by name; describe more complex techniques in the Methods section.*
☐	☒	A description of all covariates tested
☐	☒	A description of any assumptions or corrections, such as tests of normality and adjustment for multiple comparisons
☐	☒	A full description of the statistical parameters including central tendency (e.g. means) or other basic estimates (e.g. regression coefficient) AND variation (e.g. standard deviation) or associated estimates of uncertainty (e.g. confidence intervals)
☐	☒	For null hypothesis testing, the test statistic (e.g. *F*, *t*, *r*) with confidence intervals, effect sizes, degrees of freedom and *P* value noted<br>*Give P values as exact values whenever suitable.*
☒	☐	For Bayesian analysis, information on the choice of priors and Markov chain Monte Carlo settings
☒	☐	For hierarchical and complex designs, identification of the appropriate level for tests and full reporting of outcomes
☒	☐	Estimates of effect sizes (e.g. Cohen's *d*, Pearson's *r*), indicating how they were calculated

Our web collection on statistics for biologists contains articles on many of the points above.

Software and code

Policy information about availability of computer code

Data collection	BD FACS Diva 8.0.1 software; HKL3000 v716.1; Phenix-1.17.1-3660; Coot 0.9.3 EL; PyMOL 1.6.0.0; GraphPad Prism Version 8.0; Biacore X100 Evaluation Software (version: 2.0.1); ImageJ software 2.0.0-rc-69/1.52p
Data analysis	FACS data were analyzed using BD FACS Diva 8.0.1 software. The X-ray data set on the N protein and mAb nCoV396 complex was indexed and integrated using the program HKL3000 v716.1 and the structure was solved using the structure of SARS-2-N Protein NTD(PDB:6M3M) and monoclonal antibody omalizumab Fab (PDB:6TCN) as search models during molecular replacement carried out using the program Phaser as implemented in the Phenix-1.17.1-3660.The model building was carried out manually by using the program Coot 0.9.3 EL. All figures were prepared by PyMOL 1.6.0.0.. Fluorescent Quenched Substrate Assays raw data were calculated by GraphPad Prism Version 8.0 computer program. SPR data were analyzed using Biacore X100 Evaluation Software (version: 2.0.1). Image J was used for grayscale analysis of Electrophoretic mobility shift assays.

For manuscripts utilizing custom algorithms or software that are central to the research but not yet described in published literature, software must be made available to editors and reviewers. We strongly encourage code deposition in a community repository (e.g. GitHub). See the Nature Research guidelines for submitting code & software for further information.

Data

Policy information about availability of data

All manuscripts must include a data availability statement. This statement should provide the following information, where applicable:

- Accession codes, unique identifiers, or web links for publicly available datasets
- A list of figures that have associated raw data
- A description of any restrictions on data availability

Data generated or analyzed during this study are included in this published article (and its supplementary information files). Source data are provided with this

paper. Any other raw data pertaining to this study are available from the corresponding author upon reasonable request. The coordinates and structure factors files for the complex of SARS-CoV-2 N-NTD with nCoV396 was deposited to Protein Data Bank with accession code 7CR5.

Field-specific reporting

Please select the one below that is the best fit for your research. If you are not sure, read the appropriate sections before making your selection.

[x] Life sciences [ ] Behavioural & social sciences [ ] Ecological, evolutionary & environmental sciences

For a reference copy of the document with all sections, see nature.com/documents/nr-reporting-summary-flat.pdf

Life sciences study design

All studies must disclose on these points even when the disclosure is negative.

Sample size	For antibodies isolation, 6 patients with confirmed COVID-19 and a healthy donor (non-COVID-19) as control; for fluorescence-quenched substrate assays, 13 donors with abnormal or normal serologic C3 values.
Data exclusions	No data was excluded from analysis.
Replication	ELISA and the fluorescent quenched substrate assays were performed in triplicates. And we performed n=6 biologically independent COVID-19 convalescent donors in ELISA. For fluorescent quenched substrate assays, ten biologically independent abnormal C3 serum samples (n=10) and three normal samples (n=3) were analyzed in reaction systems. All attempts at replication were successful.
Randomization	We used simple random sampling to choose COVID-19 convalescent donors and donors with abnormal or normal serologic C3 in the Fifth Affiliated Hospital of Sun Yat-sen University, China.
Blinding	Blinding is not relevant to this study because sample identities (COVID-19 convalescents, donors with abnormal or normal serologic C3 values) were known during samples collection.

Reporting for specific materials, systems and methods

We require information from authors about some types of materials, experimental systems and methods used in many studies. Here, indicate whether each material, system or method listed is relevant to your study. If you are not sure if a list item applies to your research, read the appropriate section before selecting a response.

Materials & experimental systems

n/a	Involved in the study	
[ ]	[x]	Antibodies
[ ]	[x]	Eukaryotic cell lines
[x]	[ ]	Palaeontology and archaeology
[x]	[ ]	Animals and other organisms
[ ]	[x]	Human research participants
[x]	[ ]	Clinical data
[x]	[ ]	Dual use research of concern

Methods

n/a	Involved in the study	
[x]	[ ]	ChIP-seq
[ ]	[x]	Flow cytometry
[x]	[ ]	MRI-based neuroimaging

Antibodies

Antibodies used	Sorting of single plasma cells and memory B cells by FACS, CD3-PE-Cy5(PE-Cy™5 Mouse Anti-Human CD3, BD Pharmingen™, cat. 555334, clone UCHT1, lot. 0037612, 1:50 dilution), CD14-FITC(Mouse anti-human CD14-FITC, BD Pharmingen™, cat. 555397, clone M5E2, lot. 0057235, 1:10 dilution), CD14-PE-Cy5(CD14 Monoclonal Antibody, Bioscience™, cat. 25-0149-42, clone 61D3, lot. 4291431, 1:200 dilution), CD16-PE-Cy5(PE-Cy™5 Mouse Anti-Human CD16, BD Pharmingen™, cat. 555408, clone 3G8, lot. 0002965, 1:50 dilution), CD235a-PE(PE Mouse Anti-Human glycophorin A, BD biosciences, cat. 340947, clone GAR-2(HIR-2), lot. 5124620, 1:200 dilution), CD20-APC(APC Mouse Anti-Human CD20, BD Pharmingen™, clone 2H7, cat. 559776, lot. 80005977, 1:40 dilution), CD19-APC-H7(APC-H7 Mouse Anti-Human CD19, BD Pharmingen™, cat. 560727, clone. HIB19, lot. 0268687, 1:50 dilution), CD27-BV421 (BV421 Mouse Anti-Human CD27, BD Horizon™, cat. 562513, clone M-T271, lot. 9338698, 1:50 dilution), CD27-APC-H7(APC-H7 Mouse Anti-Human CD27, BD Pharmingen™, cat. 560222, clone M-T271, lot. 9303874, 1:50 dilution), IgD-PE(PE Mouse Anti-Human IgD, BD Pharmingen™, cat. 555779, clone IA6-2, lot. 7325981, 1:40 dilution) and CD38-APC(APC Mouse Anti-Human CD38, BD Pharmingen™, cat. 555462, clone HIT2, lot. 0016328, 1:10 dilution) antibodies were used. And Goat anti-human IgG-horseradish peroxidase(Goat anti-human IgG-horseradish peroxidase, Promega Corporation, cat. W4031, lot. 0000424299, 1:10000 dilution) was used as secondary antibody for ELISA.
Validation	All the antibodies used in this study were commercial antibodies and were only used for applications, with validation procedures described on the following sites of the manufacturers:<br>CD3-PE-Cy5(PE-Cy™5 Mouse Anti-Human CD3, BD Pharmingen™, cat. 555334, clone UCHT1, lot. 0037612, 1:50 dilution) https://www.bdbiosciences.com/us/applications/research/t-cell-immunology/th-1-cells/surface-markers/human/pe-cy5-mouse-anti-human-

cd3-ucht1-also-known-as-ucht-1-ucht-1/p/555334
CD14-FITC(Mouse anti-human CD14-FITC, BD Pharmingen™, cat. 555397, clone M5E2, lot. 0057235, 1:10 dilution)
https://www.bdbiosciences.com/us/applications/research/stem-cell-research/hematopoietic-stem-cell-markers/human/negative-markers/fitc-mouse-anti-human-cd14-m5e2/p/555397
CD14-PE-Cy5(CD14 Monoclonal Antibody, Bioscience™, cat. 25-0149-42, clone 61D3, lot. 4291431, 1:200 dilution)
https://www.thermofisher.com/cn/zh/antibody/product/CD14-Antibody-clone-61D3-Monoclonal/25-0149-42
CD16-PE-Cy5(PE-Cy™5 Mouse Anti-Human CD16, BD Pharmingen™, cat. 555408, clone 3G8, lot. 0002965, 1:50 dilution)
https://www.bdbiosciences.com/eu/applications/research/stem-cell-research/cancer-research/human/pe-cy5-mouse-anti-human-cd16-3g8/p/555408
CD235a-PE(PE Mouse Anti-Human glycophorin A, BD biosciences, cat. 340947, clone GAR-2(HIR-2), lot. 5124620, 1:200 dilution)
https://www.bdbiosciences.com/us/reagents/research/clinical-research---ruo-gmp/single-color-antibodies/pe-mouse-anti-human-glycophorin-a-ga-r2-hir2/p/340947
CD20-APC(APC Mouse Anti-Human CD20, BD Pharmingen™, clone 2H7, cat. 559776, lot. 80005977, 1:40 dilution)
https://www.bdbiosciences.com/us/applications/research/stem-cell-research/hematopoietic-stem-cell-markers/human/negative-markers/apc-mouse-anti-human-cd20-2h7/p/559776
CD19-APC-H7(APC-H7 Mouse Anti-Human CD19, BD Pharmingen™, cat. 560727, clone. HIB19, lot. 0268687, 1:50 dilution)
https://www.bdbiosciences.com/us/applications/research/stem-cell-research/hematopoietic-stem-cell-markers/human/negative-markers/apc-h7-mouse-anti-human-cd19-hib19/p/560727
CD27-BV421(BV421 Mouse Anti-Human CD27, BD Horizon™, cat. 562513, clone M-T271, lot. 9338698, 1:50 dilution)
https://www.bdbiosciences.com/us/applications/research/clinical-research/oncology-research/blood-cell-disorders/surface-markers/human/bv421-mouse-anti-human-cd27-m-t271/p/562513
CD27-APC-H7(APC-H7 Mouse Anti-Human CD27, BD Pharmingen™, cat. 560222, clone M-T271, lot. 9303874, 1:50 dilution)
https://www.bdbiosciences.com/us/applications/research/clinical-research/oncology-research/blood-cell-disorders/surface-markers/human/apc-h7-mouse-anti-human-cd27-m-t271/p/560222
IgD-PE(PE Mouse Anti-Human IgD, BD Pharmingen™, cat. 555779, clone IA6-2, lot. 7325981, 1:40 dilution)
https://www.bdbiosciences.com/us/applications/research/b-cell-research/immunoglobulins/human/pe-mouse-anti-human-igd-ia6-2-also-known-as--ia6-2/p/555779
CD38-APC(APC Mouse Anti-Human CD38, BD Pharmingen™, cat. 555462, clone HIT2, lot. 0016328, 1:10 dilution)
https://www.bdbiosciences.com/us/applications/research/t-cell-immunology/regulatory-t-cells/surface-markers/human/apc-mouse-anti-human-cd38-hit2/p/555462

Eukaryotic cell lines

Policy information about cell lines

Cell line source(s)	Expi293F™(ThermoFisher) and 293T(ATCC)
Authentication	None of the cell lines used were authenticated
Mycoplasma contamination	All cell lines used were tested and were mycoplasma free
Commonly misidentified lines (See ICLAC register)	No commonly misidentified lines were used in this study

Human research participants

Policy information about studies involving human research participants

Population characteristics	For COVID-19 convalescents donors(age 30-66; mean 46), blood samples were collected 9 - 25 days after the onset of the disease. The fluorescent quenched substrate assays enrolled 13 donors (age 7-63; mean 42.5) with abnormal or normal serologic C3 values.
Recruitment	For antibodies isolations, eligible participants were patients admitted to the Fifth Affiliated Hospital, Sun Yat-sen University with RT-PCR-confirmed SARS-CoV-2. For fluorescence-quenched substrate assays, the donors with abnormal or normal serologic C3 values were recruited. All samples were recruited from random selection. There was no potential self-selection bias or other biases during the selection.
Ethics oversight	This study received approval from the Research Ethics Committee of the Fifth Affiliated Hospital of Sun Yat-sen University, China (approval number:K198-1), and all participants provided written informed consent.

Note that full information on the approval of the study protocol must also be provided in the manuscript.

Flow Cytometry

Plots

Confirm that:

- [x] The axis labels state the marker and fluorochrome used (e.g. CD4-FITC).
- [x] The axis scales are clearly visible. Include numbers along axes only for bottom left plot of group (a 'group' is an analysis of identical markers).
- [x] All plots are contour plots with outliers or pseudocolor plots.
- [x] A numerical value for number of cells or percentage (with statistics) is provided.

Methodology

Sample preparation	PBMC and plasma were isolated from bloods by Ficoll-Paque PLUS (GE,17-1440-02) density gradient centrifugation. Then PBMC stain for 30 minutes at 25°C and avoid light, wash twice with PBS, and resuspend in PBS.
Instrument	BD FACS Aria SORP
Software	BD FACS Diva 8.0.1
Cell population abundance	Plasma cells sorted 240 Single cells and memory B cells sorted 76 Single cells.
Gating strategy	For plasma cell sort: FSC-A, SSC-A gate lymphocytes, FSC-W, FSC-H and SSC-W, SSC-H gate singlets, Aque vital-AmCyan negative gate live cells, CD3neg/CD14neg/CD16neg/CD19pos gate total B cells, IgDneg gate mature B cells, CD27high/CD38high gate plasma cells. For specific B cell sort: FSC-A, SSC-A gate lymphocytes, FSC-W, FSC-H and SSC-W, SSC-H gate singlets, Aque vital-AmCyan negative gate live cells, CD3neg/CD14neg/CD16neg/CD20pos gate total B cells, IgDneg/CD27pos gate memory B cells, Antigen-BV421pos/Antigen-PE-Cy7pos gate specific B cells.

- [x] Tick this box to confirm that a figure exemplifying the gating strategy is provided in the Supplementary Information.
